# Supplementary material for: A haplotype-resolved, chromosome-scale genome for Malus domestica Borkh. ‘WA 38’
Source: G3 (Bethesda). 2024 Sep 17;14(12):jkae222. doi: 10.1093/g3journal/jkae222 (PMC11631450; doi:10.1093/g3journal/jkae222)
Supplement: jkae222_Supplementary_Data [file jkae222_supplementary_data.zip › Supplemental_Figures_G3-2024-405155.pdf]

Figure S1. Workflow of the project and each step, including A) summary workflow, B) quality control workflow, C) nuclear assembly workflow, D) Structural annotation workflow, E) Function Annotation workflow, F) Comparative Analysis Workflow, G) Organelle Assembly & Annotation Workflow

A) Summary Workflow

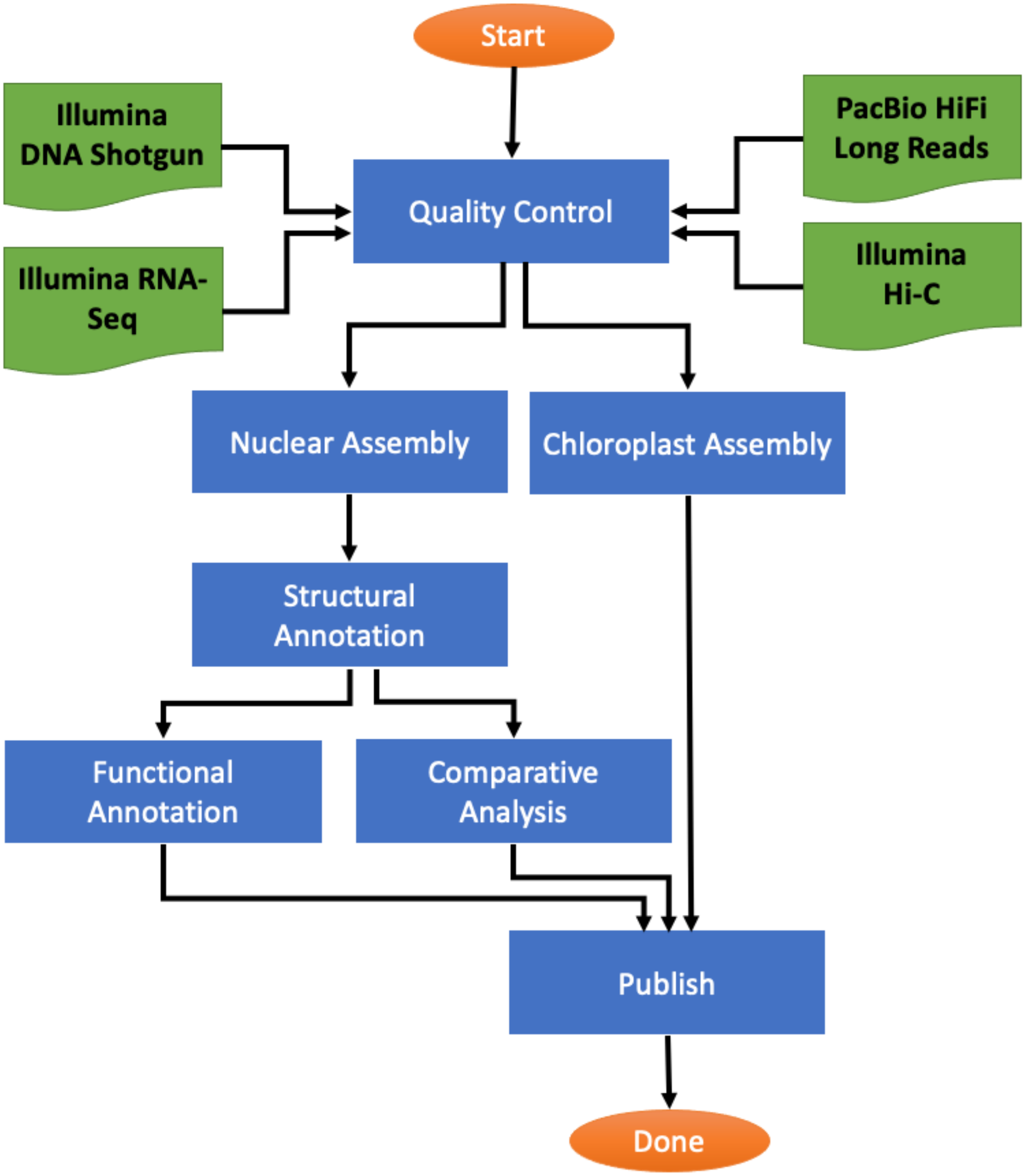

B) Quality Control Workflow

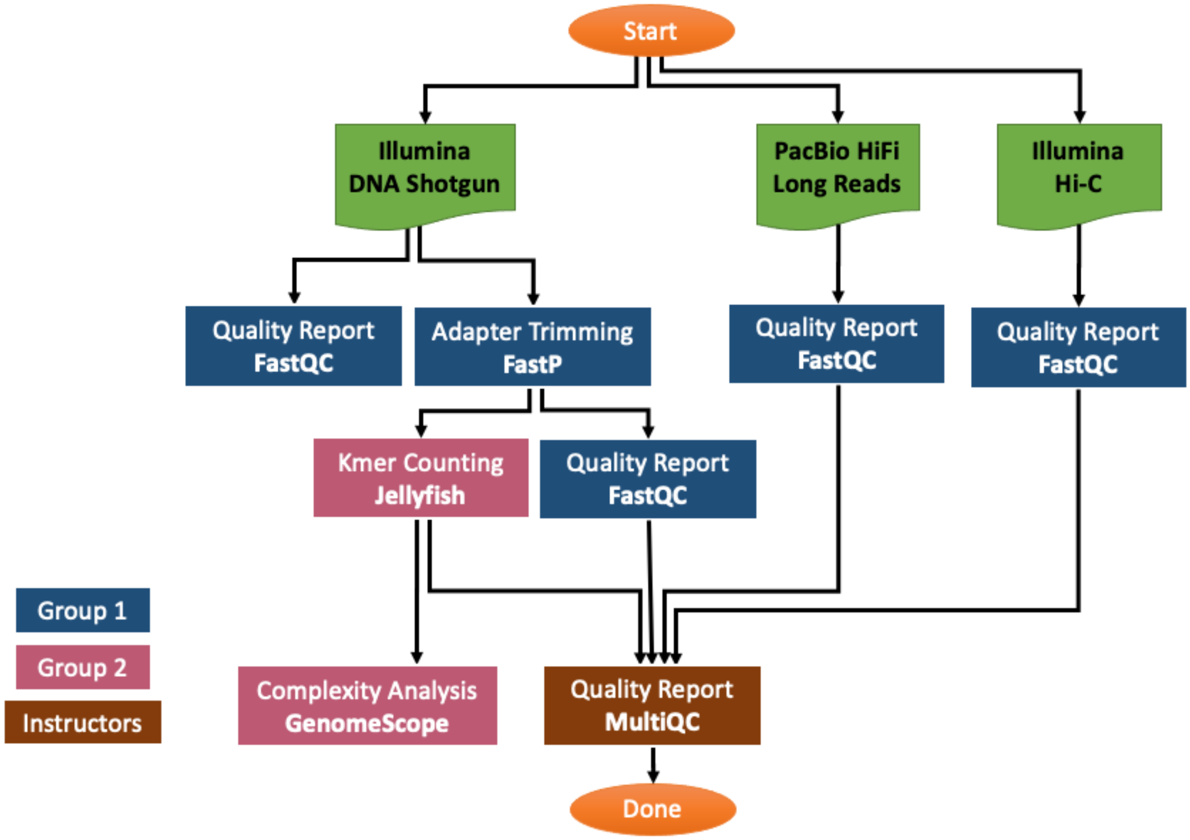

C) Nuclear Assembly Workflow

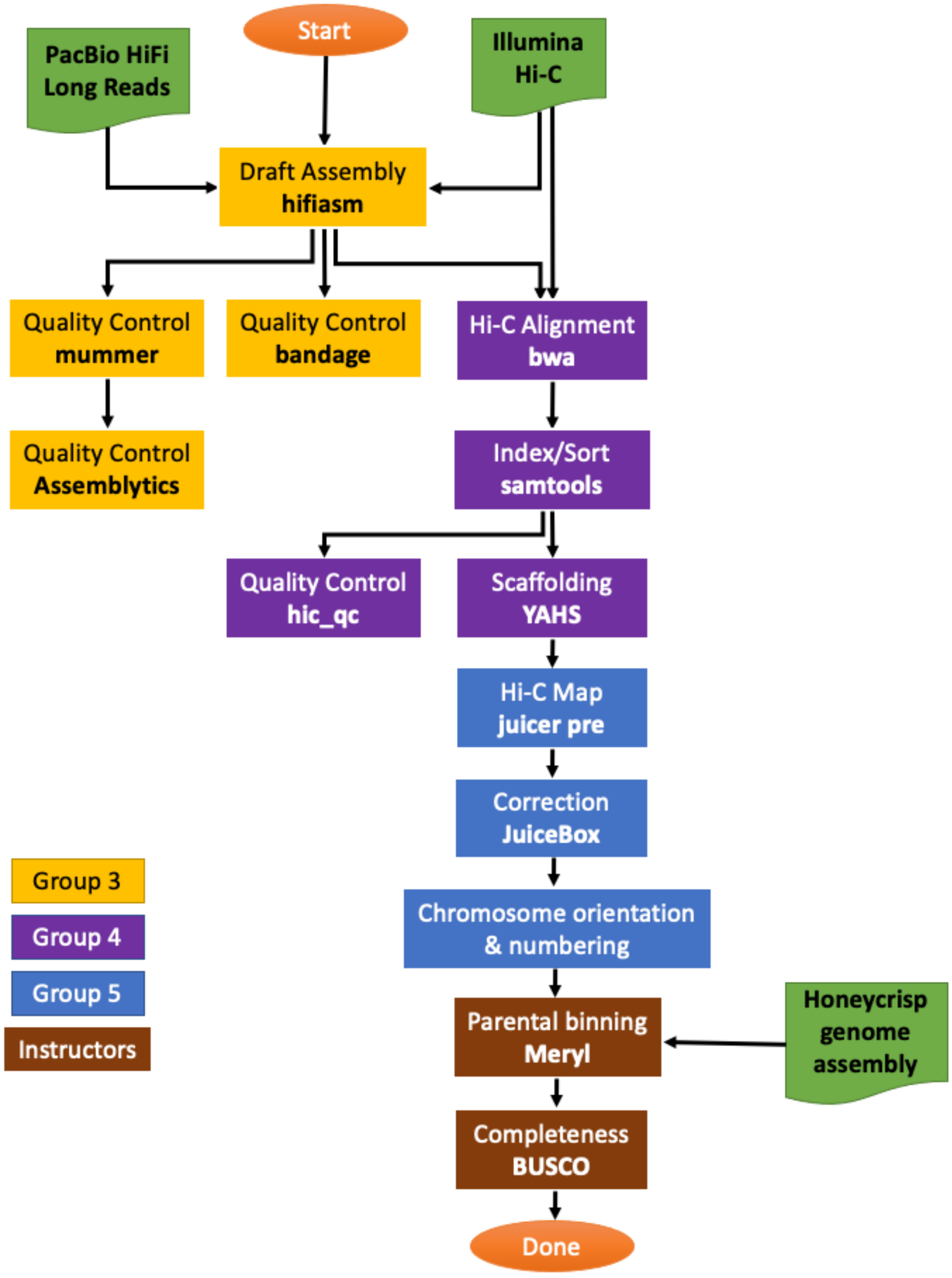

D) Structural Annotation workflow

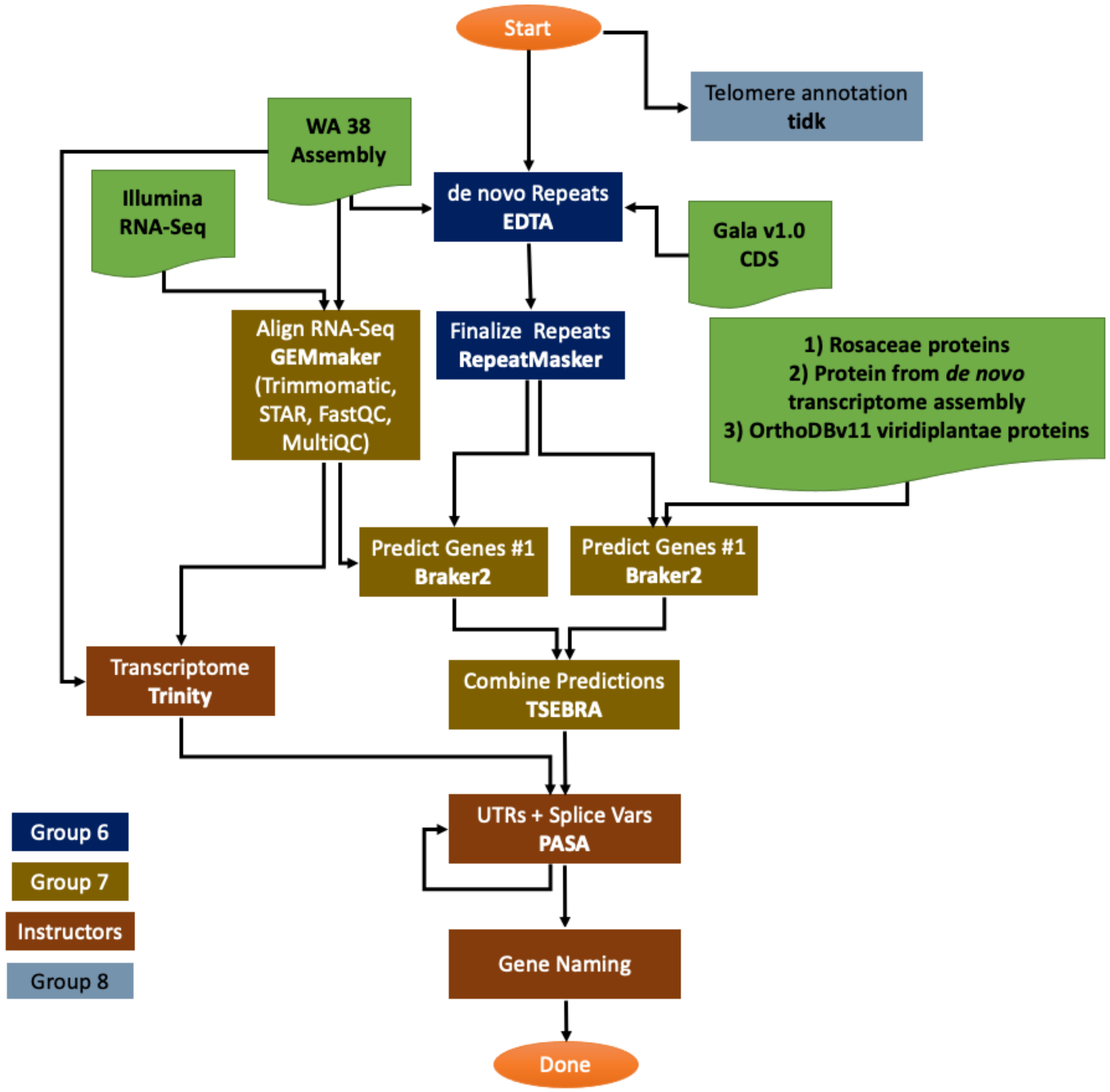

Note: Group 8 consists of students volunteered to continue working on the genome project after the class ends.

E) Function Annotation workflow

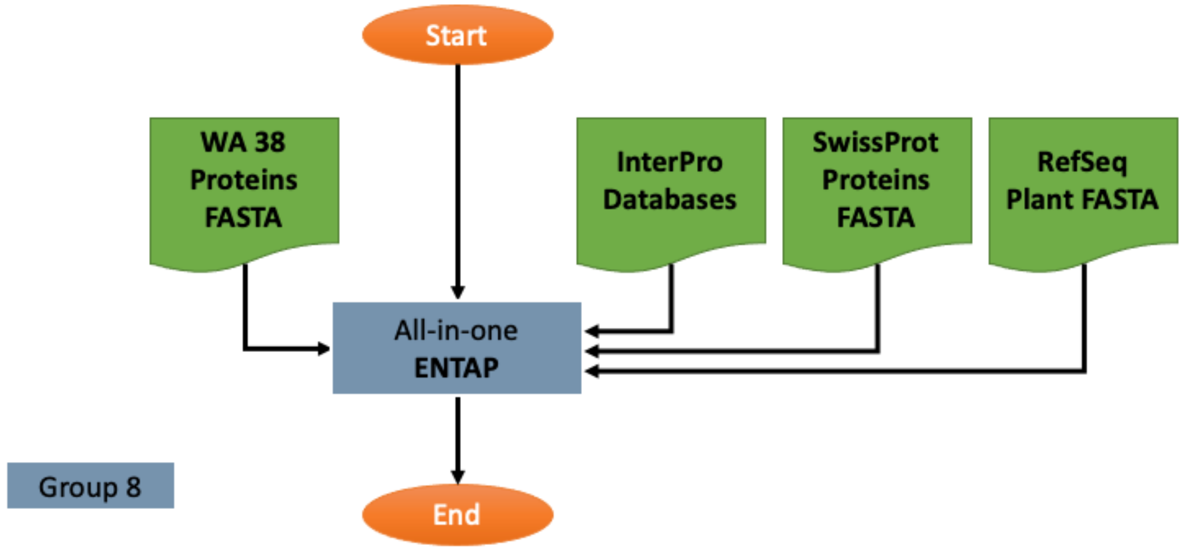

F) Comparative Analysis Workflow

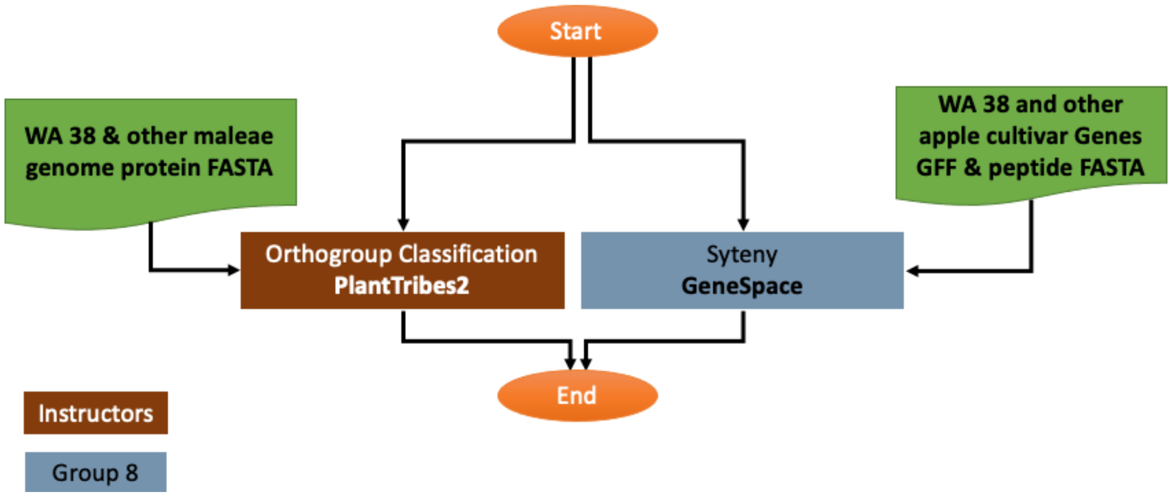

G) Chloroplast Assembly & Annotation Workflow

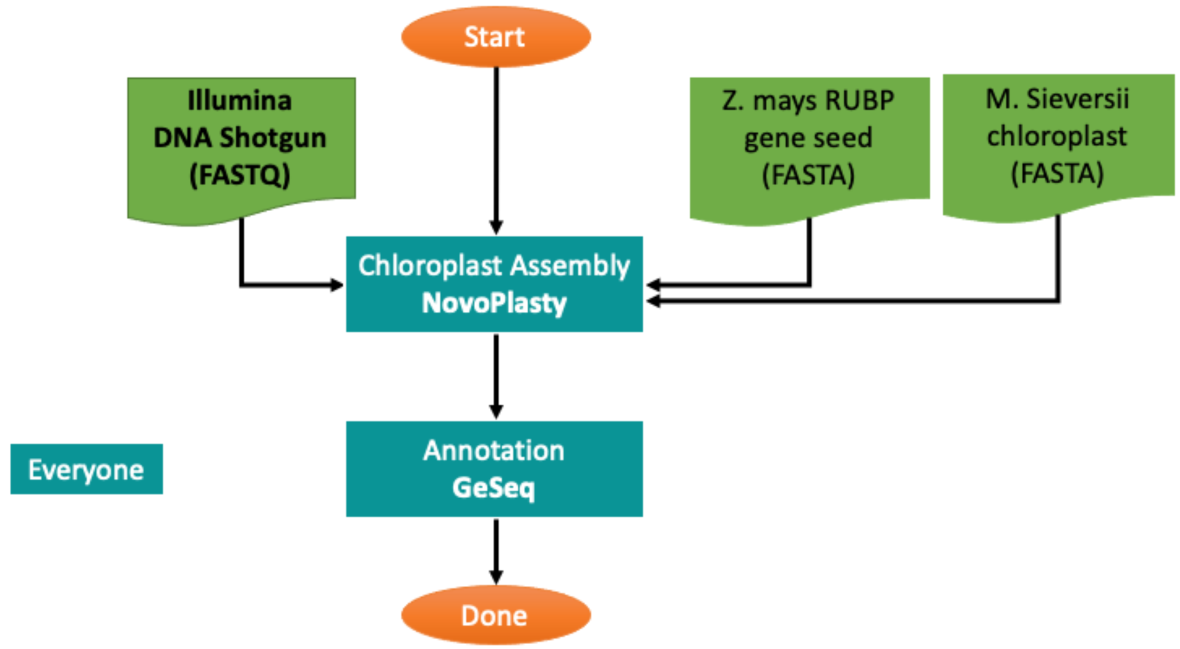

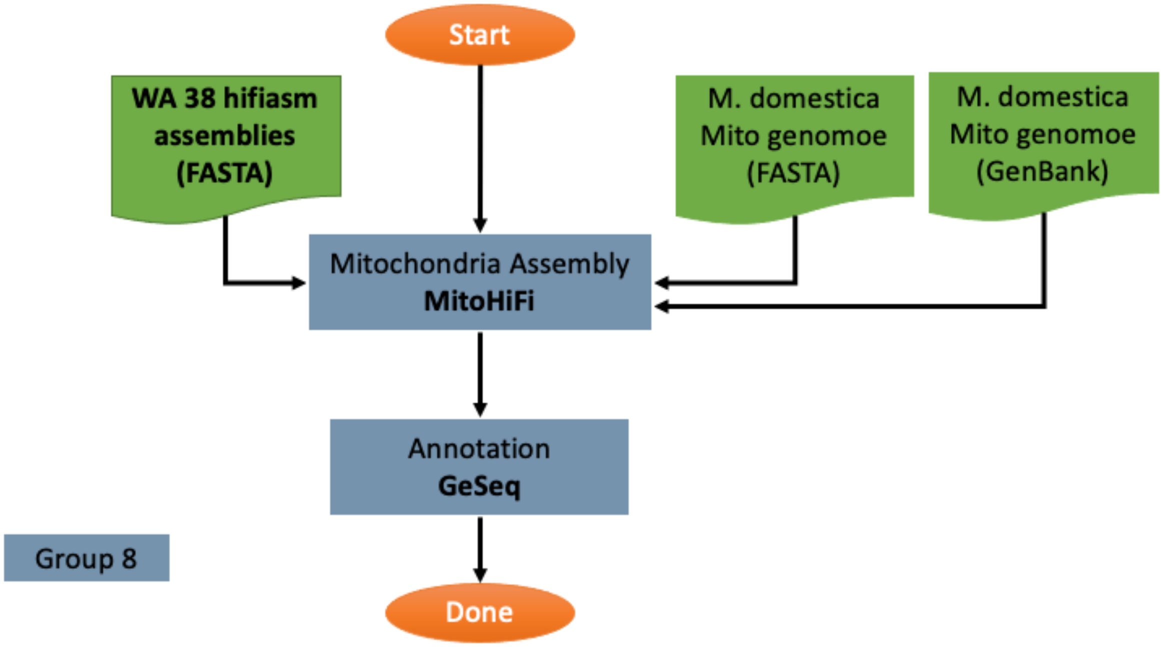

Figure S2. Chromosome re-orientation.
LASTZ alignment graph of original ‘WA 38’ chromosomes (y axis) against corresponding ‘Gala’ chromosome (x axis). Chr#A/B_cultivar indicates the sequence in the orientation, Chr#A/B_reoriented_cultivar indicates the chromosome is reoriented according to the corresponding ‘Gala’ chromosome orientation. Blue lines indicate sequences matching in the same orientation, while red lines indicates sequences matching in reverse orientation. Only straight continuous diagonal lines indicate alignment of the entire chromosome.

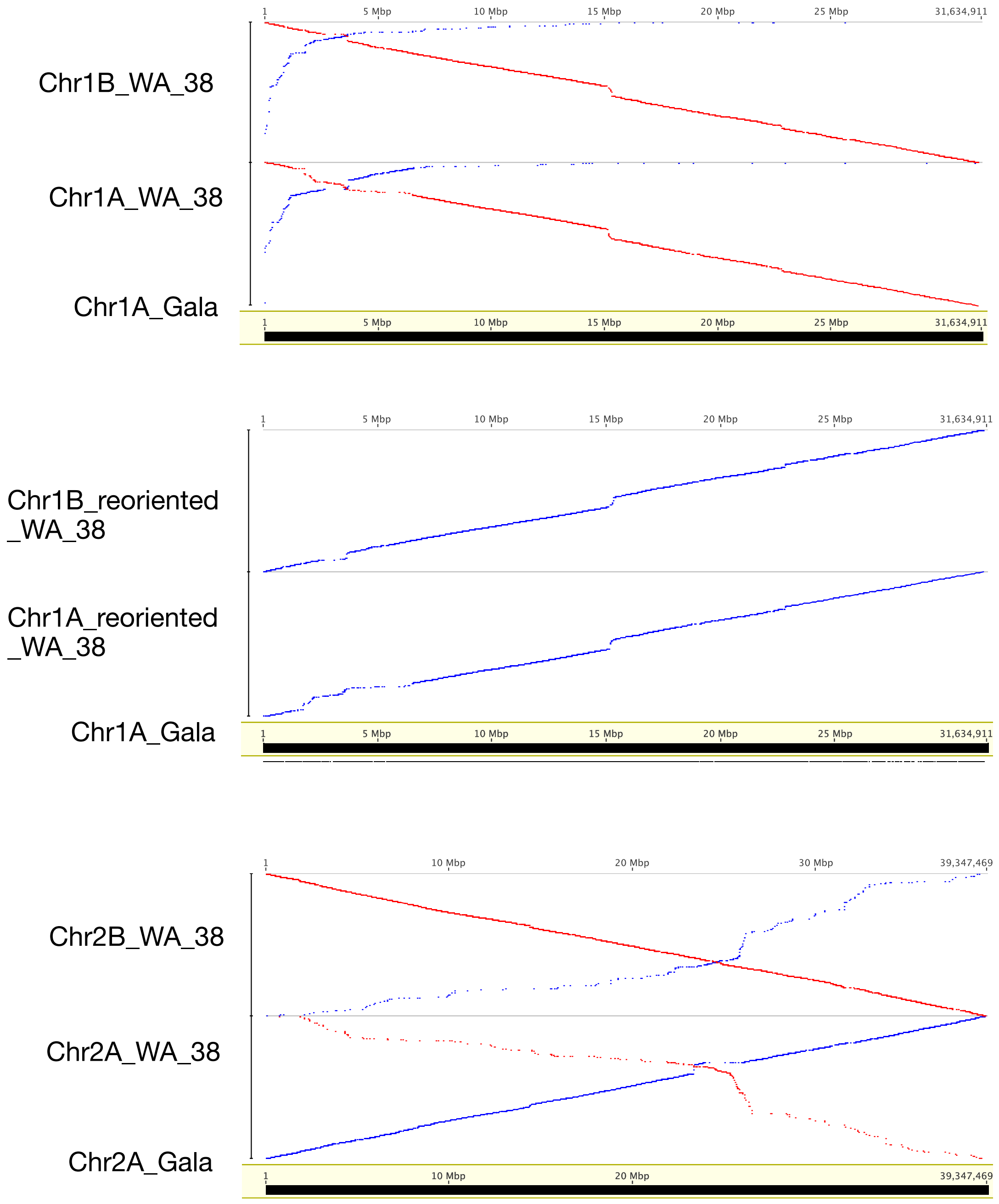

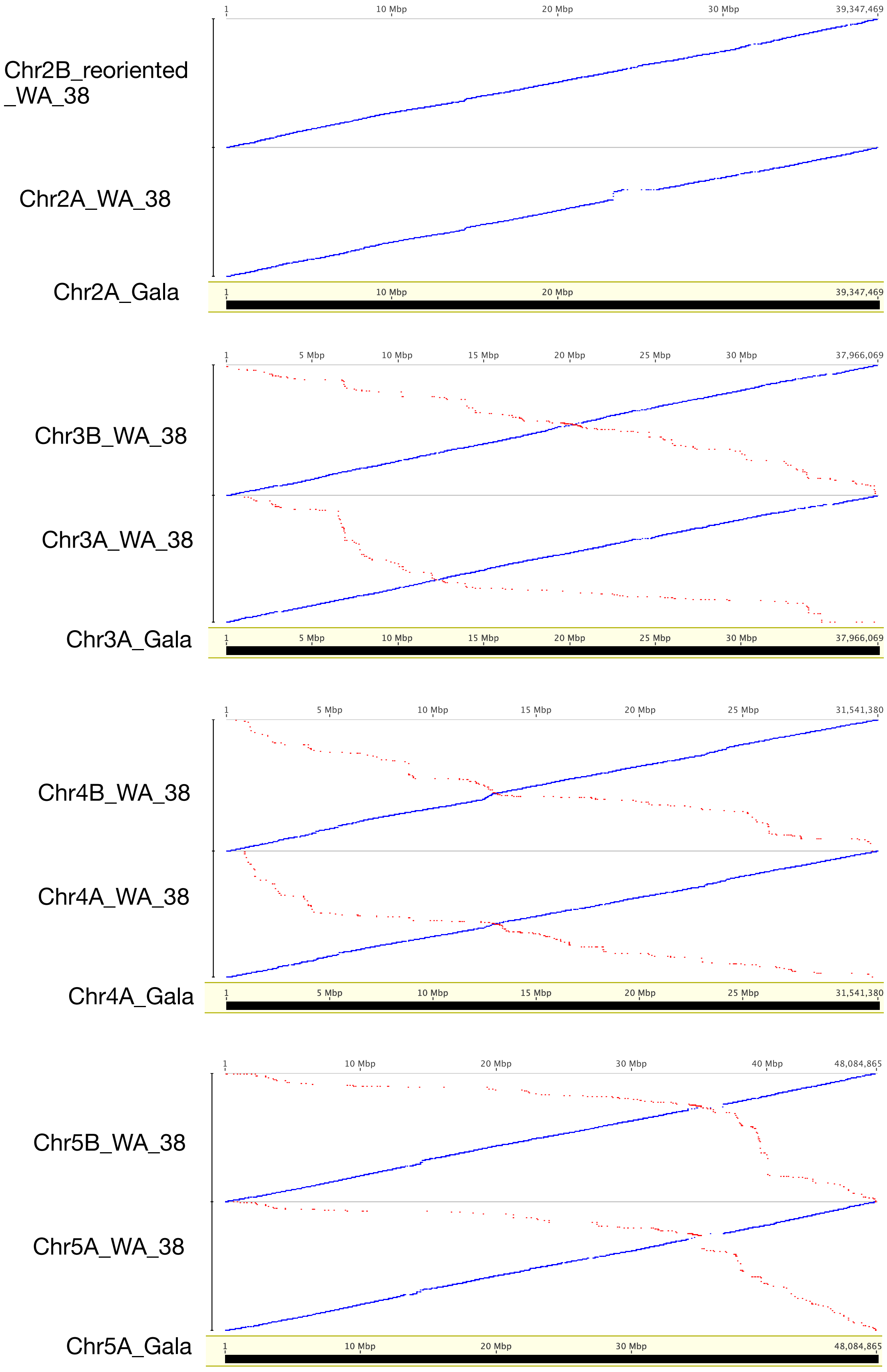
Chr2B_reoriented _WA_38
Chr2A_WA_38
Chr2A_Gala
1
10 Mbp
20 Mbp
30 Mbp
39,347,469
Chr3B_WA_38
Chr3A_WA_38
Chr3A_Gala
5 Mbp
15 Mbp
25 Mbp
37,966,069
Chr4B_WA_38
Chr4A_WA_38
Chr4A_Gala
31,541,380
Chr5B_WA_38
Chr5A_WA_38
Chr5A_Gala
40 Mbp
48,084,865

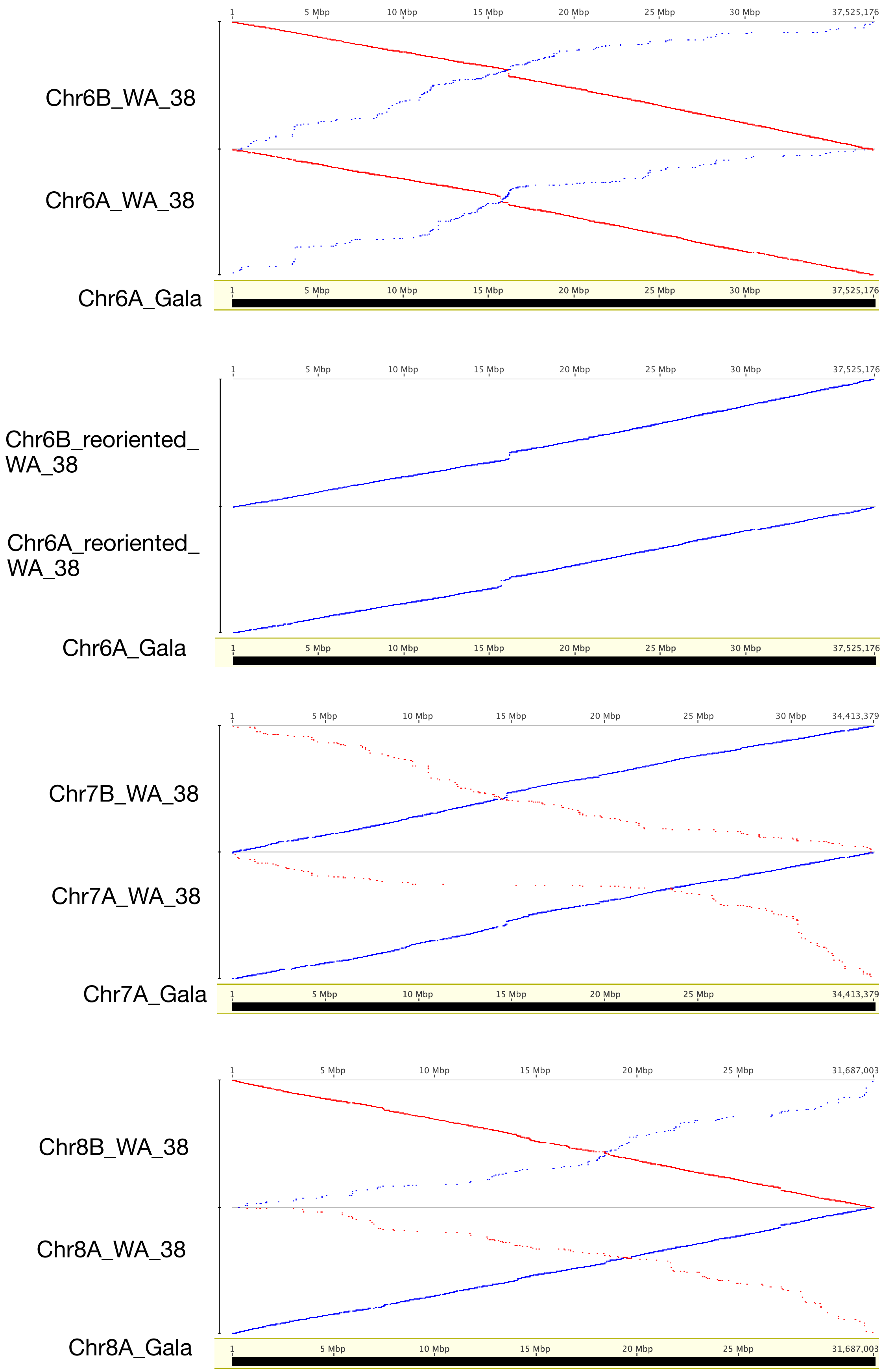
Chr6B_WA_38
Chr6A_WA_38
Chr6A_Gala
1
5 Mbp
10 Mbp
15 Mbp
20 Mbp
25 Mbp
30 Mbp
37,525,176
Chr6B_reoriented_WA_38
Chr6A_reoriented_WA_38
Chr6A_Gala
Chr7B_WA_38
Chr7A_WA_38
Chr7A_Gala
34,413,379
Chr8B_WA_38
Chr8A_WA_38
Chr8A_Gala
31,687,003

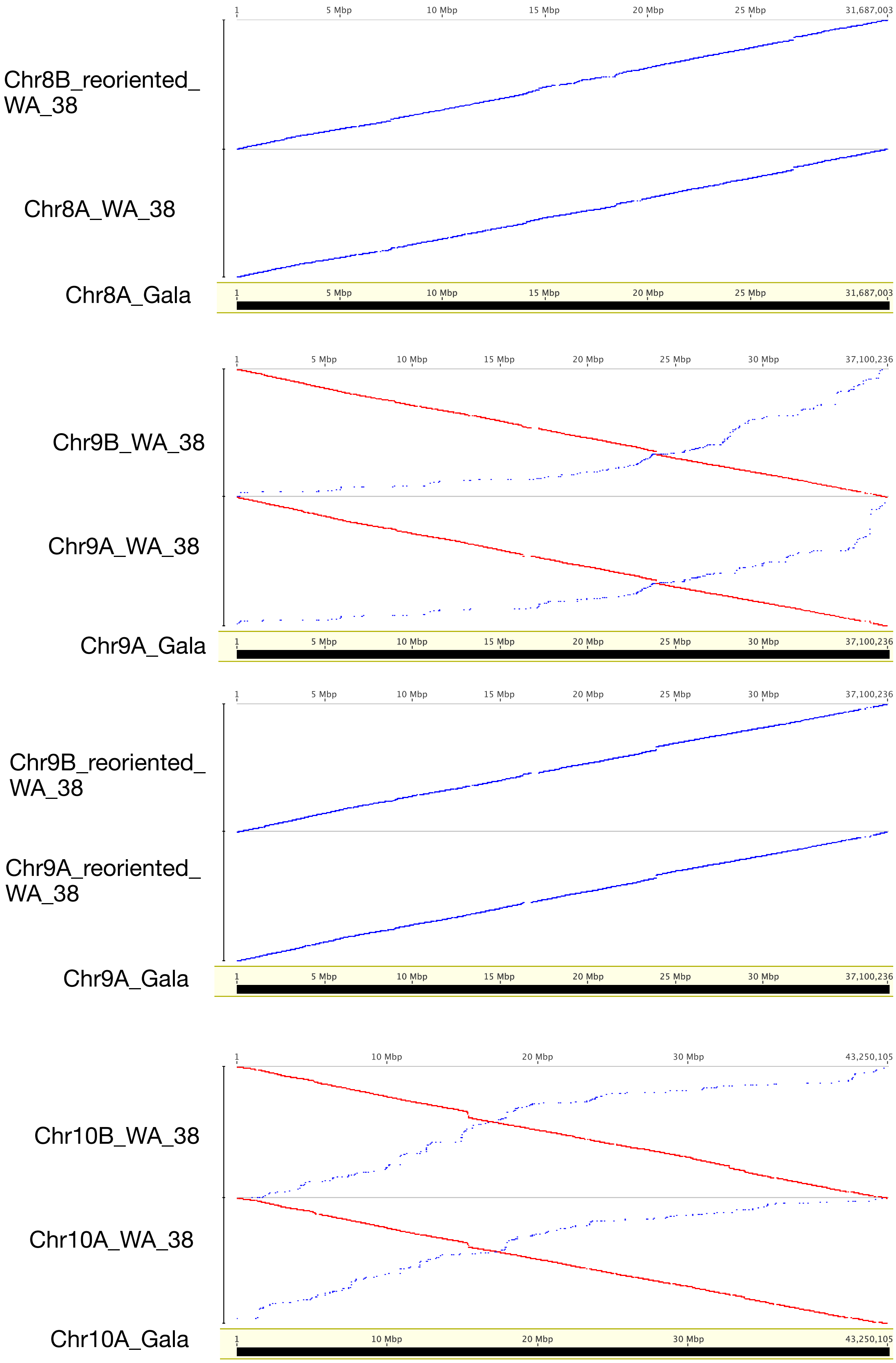
Chr8B_reoriented_WA_38
Chr8A_WA_38
Chr8A_Gala
1
5 Mbp
10 Mbp
15 Mbp
20 Mbp
25 Mbp
31,687,003
Chr9B_WA_38
Chr9A_WA_38
Chr9A_Gala
1
5 Mbp
10 Mbp
15 Mbp
20 Mbp
25 Mbp
30 Mbp
37,100,236
Chr9B_reoriented_WA_38
Chr9A_reoriented_WA_38
Chr9A_Gala
Chr10B_WA_38
Chr10A_WA_38
Chr10A_Gala
1
10 Mbp
20 Mbp
30 Mbp
43,250,105

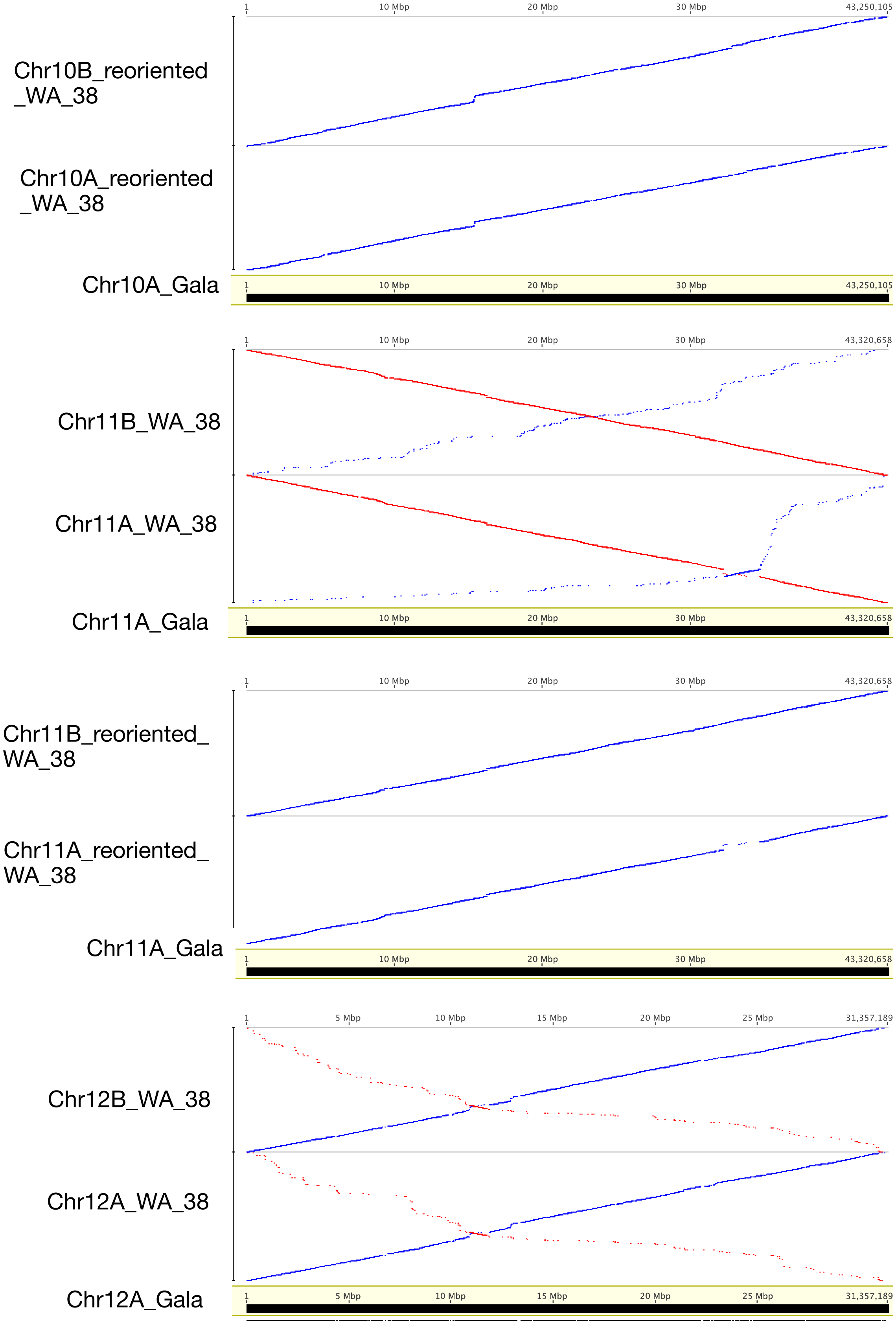
Chr10B_reoriented
_WA_38
Chr10A_reoriented
_WA_38
Chr10A_Gala
1
10 Mbp
20 Mbp
30 Mbp
43,250,105
Chr11B_WA_38
Chr11A_WA_38
Chr11A_Gala
43,320,658
Chr11B_reoriented_
WA_38
Chr11A_reoriented_
WA_38
Chr12B_WA_38
Chr12A_WA_38
Chr12A_Gala
5 Mbp
15 Mbp
25 Mbp
31,357,189

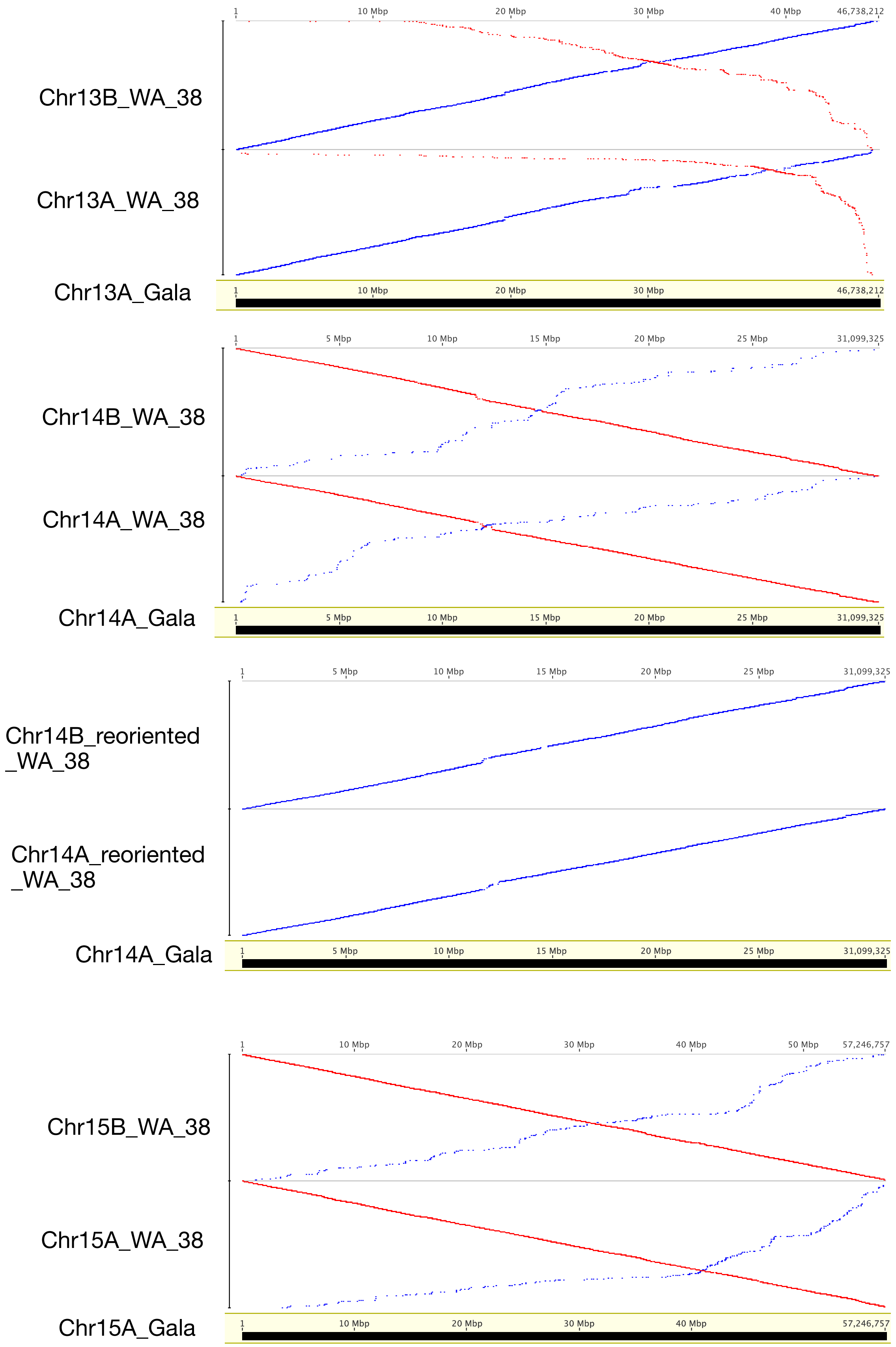
Chr13B_WA_38
Chr13A_WA_38
Chr13A_Gala
1
10 Mbp
20 Mbp
30 Mbp
40 Mbp
46,738,212
Chr14B_WA_38
Chr14A_WA_38
Chr14A_Gala
5 Mbp
10 Mbp
15 Mbp
20 Mbp
25 Mbp
31,099,325
Chr14B_reoriented_WA_38
Chr14A_reoriented_WA_38
Chr14A_Gala
Chr15B_WA_38
Chr15A_WA_38
Chr15A_Gala
50 Mbp
57,246,757

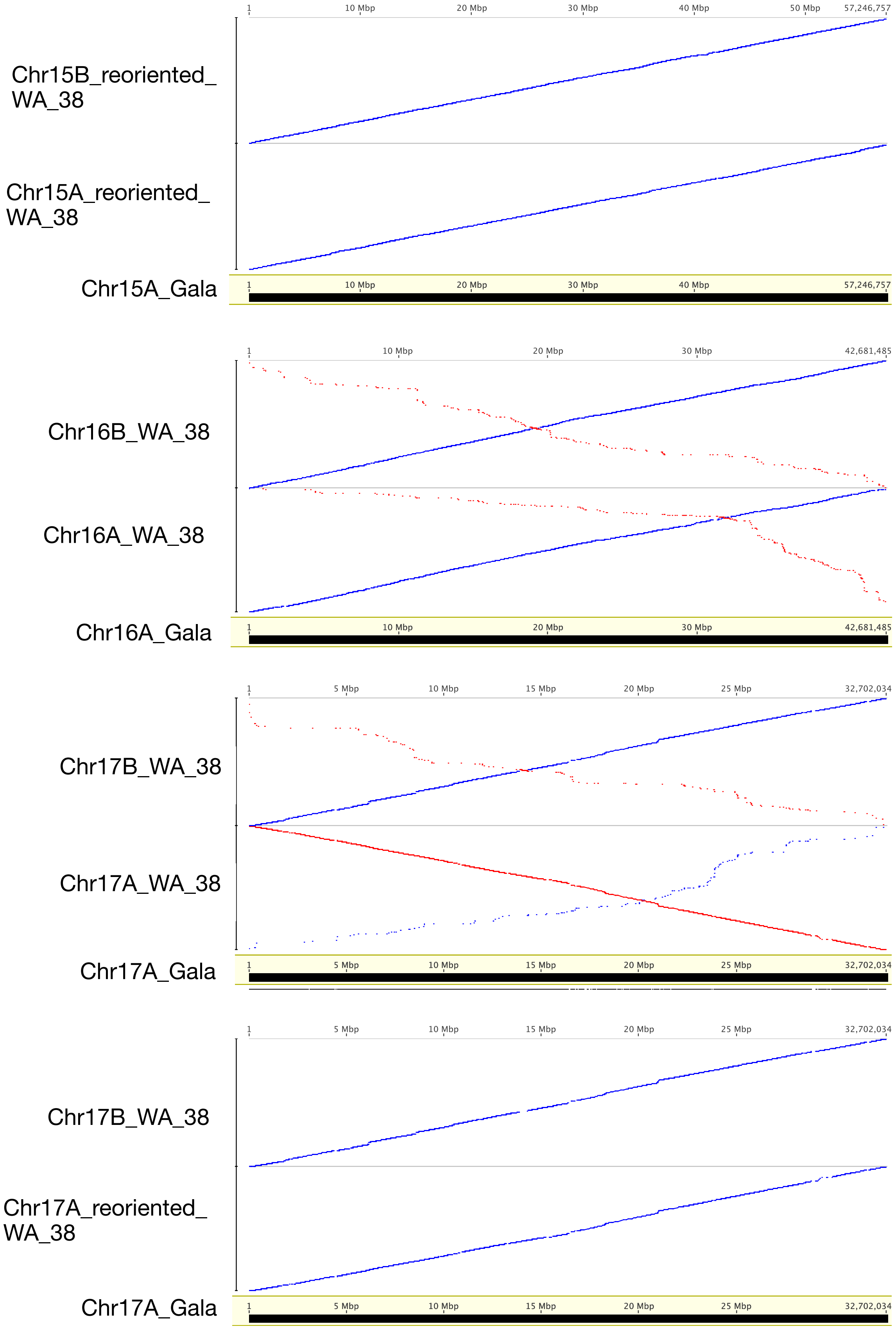

Chr15B_reoriented_WA_38
Chr15A_reoriented_WA_38
Chr15A_Gala
1
10 Mbp
20 Mbp
30 Mbp
40 Mbp
50 Mbp
57,246,757
Chr16B_WA_38
Chr16A_WA_38
Chr16A_Gala
1
10 Mbp
20 Mbp
30 Mbp
42,681,485
Chr17B_WA_38
Chr17A_WA_38
Chr17A_Gala
1
5 Mbp
10 Mbp
15 Mbp
20 Mbp
25 Mbp
32,702,034
Chr17B_WA_38
Chr17A_reoriented_WA_38
Chr17A_Gala
1
5 Mbp
10 Mbp
15 Mbp
20 Mbp
25 Mbp
32,702,034

Figure S3. Hi-C contact map for Hap1 (**A**) and Hap2 (**B**) of ‘WA 38’ genome assembly.

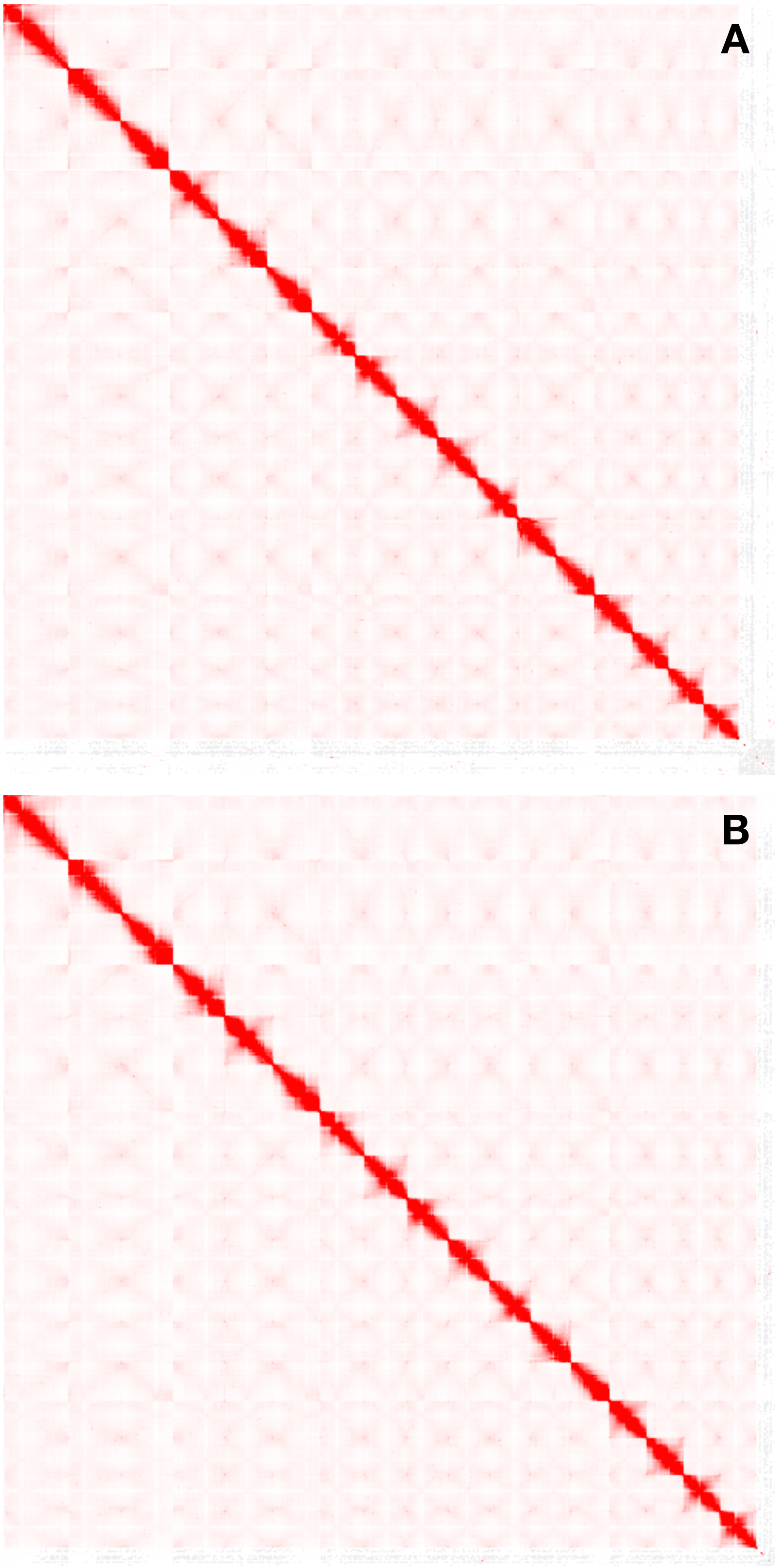

Figure S4. The assembly quality and the phasing is validated with high-quality phased 8K SNP array data of 'WA38' and corresponding genetic map. The inner and outer rings represent issues in the outcome of mapping reference SNPs to the assembly as compared to the genetic map. The height of the black bars separating the chromosomes in this ring represent 100%. Colored regions in these two rings indicate the presence of mismatching SNPs. The height of the colored region indicates the percentage of mismatches, where the width indicates the length of the affected region. Red color in these rings indicates SNPs mapped inconsistently with the genetic map whereas grey indicates unmapped SNPs. No regions with a high proportion of errors were observed. A small proportions of inconsistencies is expected and could be caused by additional polymorphisms in the SNP probe compared to 'WA38', leading to incorrect automatic determination of SNP location. Red bars in the second ring from the outer most end shows three instances of genotype inconsistency between the assembly and the reference SNP data. Upon closer investigation, such inconsistency is likely caused by incorrect (automated) location determination for these few SNPs and thus the incorrect allele is retrieved. The third ring from the outer most shows regions with greater than 5cM homozygosity according to the reference SNP array data of 'WA38', highlighted with light blue bars. The two largest rings with dark blue and cyan colors represent either paternal or maternal alleles, respectively, as determined by the phased reference SNP array data. Overall, the results from this analysis indicate highly-accurate haplotype-resolved assemblies of the 'WA 38' genome.

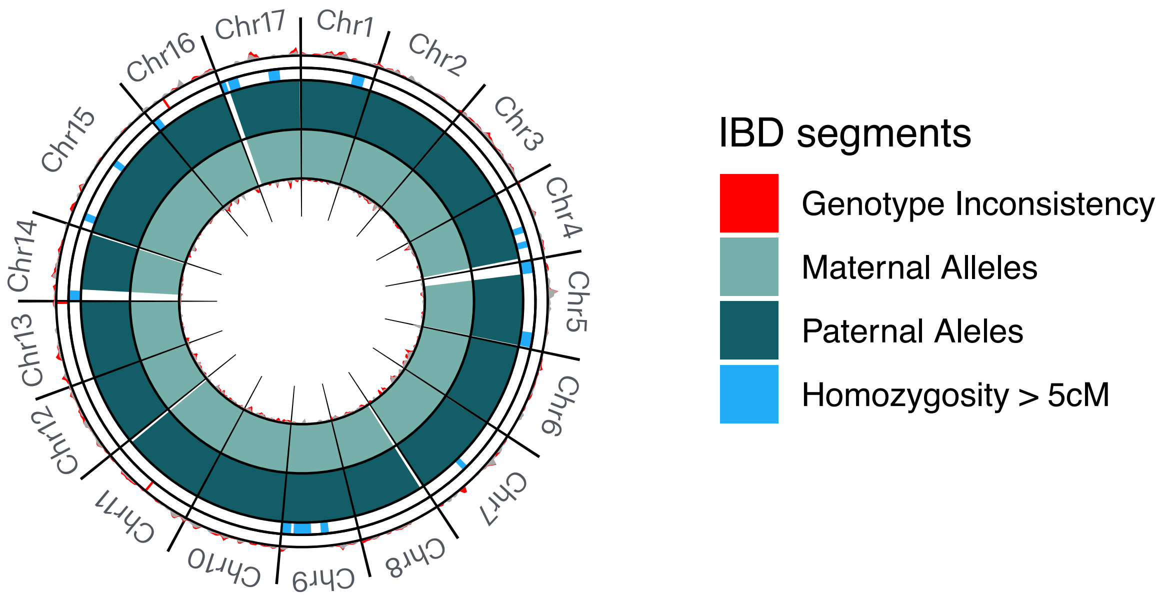

Figure S5: Structural variation between reference (WA 38 hapA) and query (WA 38 hapB). (A), Size distribution of all variants of all sizes; (B), Size distribution of variants ranging from 50-500bp; (C), Size distribution of variants ranging from 500-10,000bp; (D), Cumulative sequence length of reference and query assemblies.

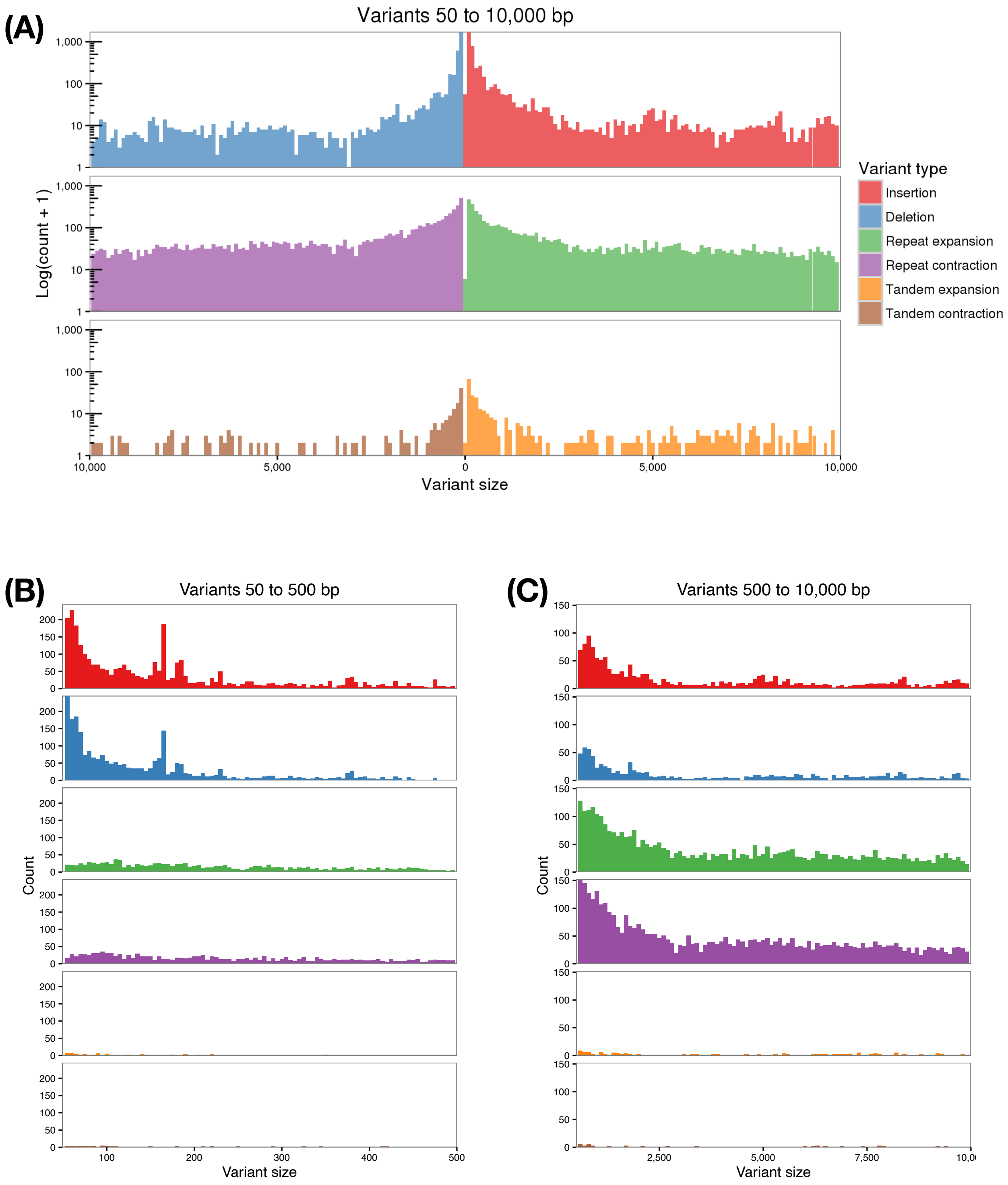

(D)

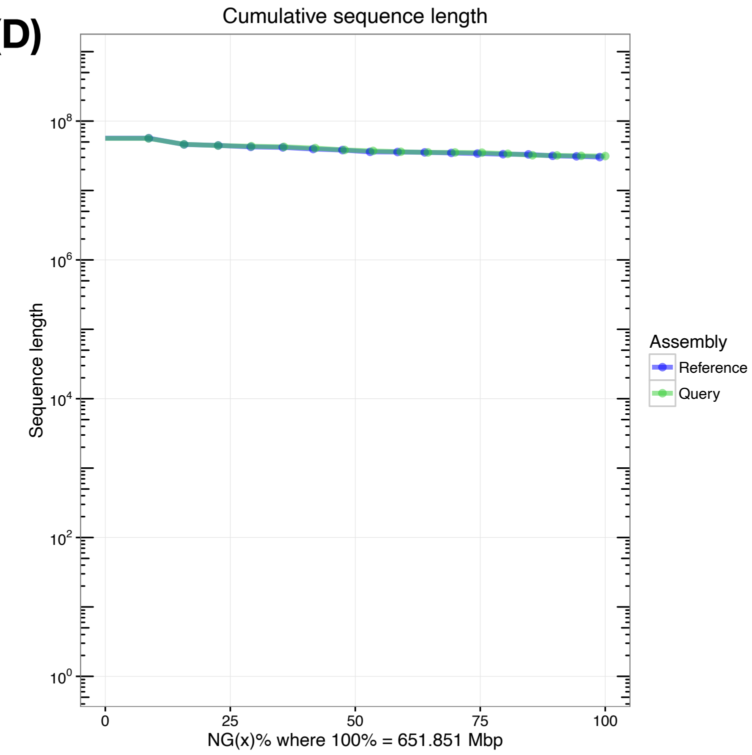

Cumulative sequence length
Sequence length
10^8
10^6
10^4
10^2
10^0
0
25
50
75
100
NG(x)% where 100% = 651.851 Mbp
Assembly
Reference
Query

Figure S6. Dot plots of genome alignment (**A**) and chromosome alignment (**B**) of the two ‘WA 38’ haplomes.

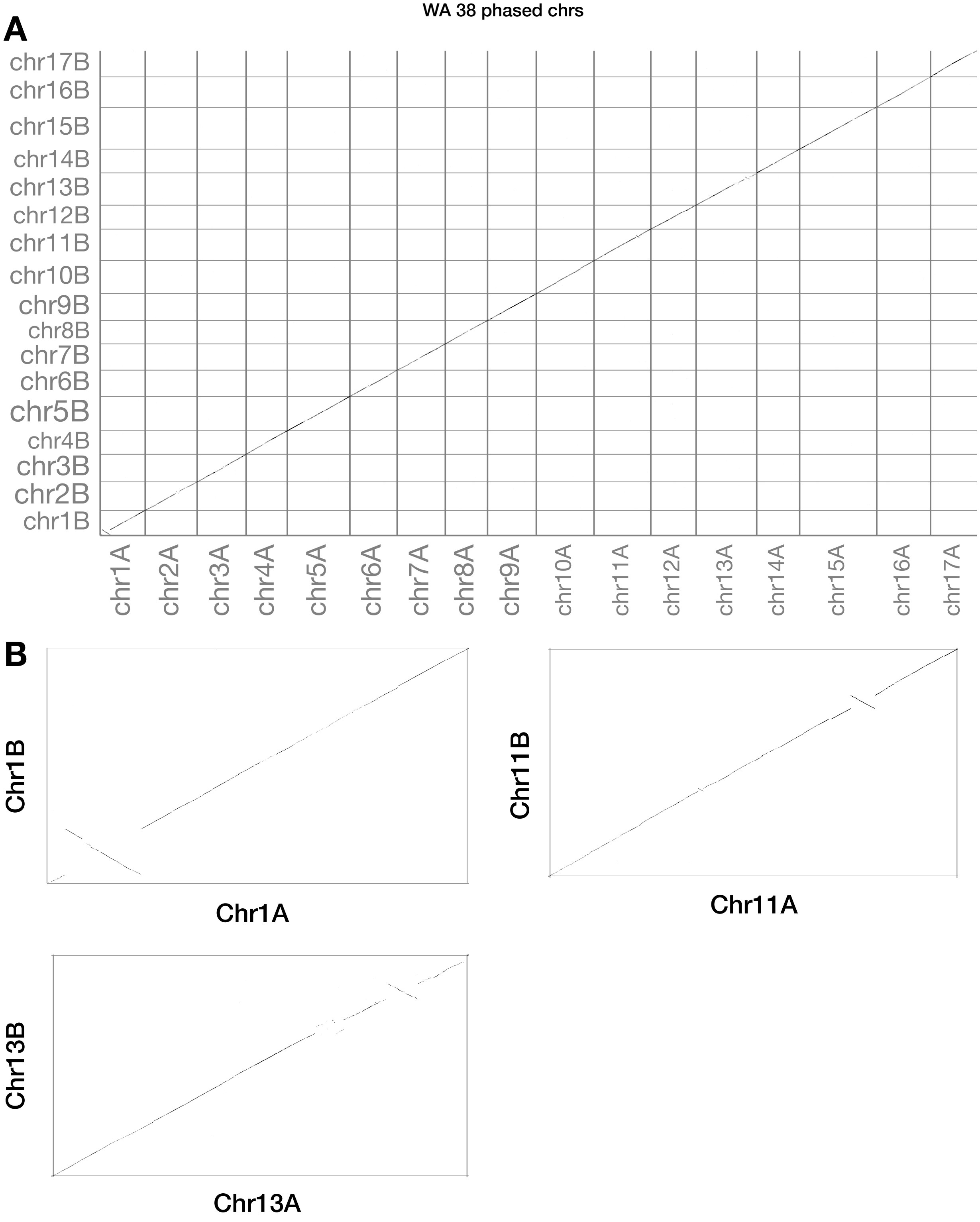

Figure S7. Issues with BRAKER gene models. (A) shows an example of gene models overlaps with repeat region and splice variants of the same gene do not overlap. (B) shows an example of overlapping gene models on the same strand. Blue lines represent gene models and mRAN, orange lines represent repeat regions.

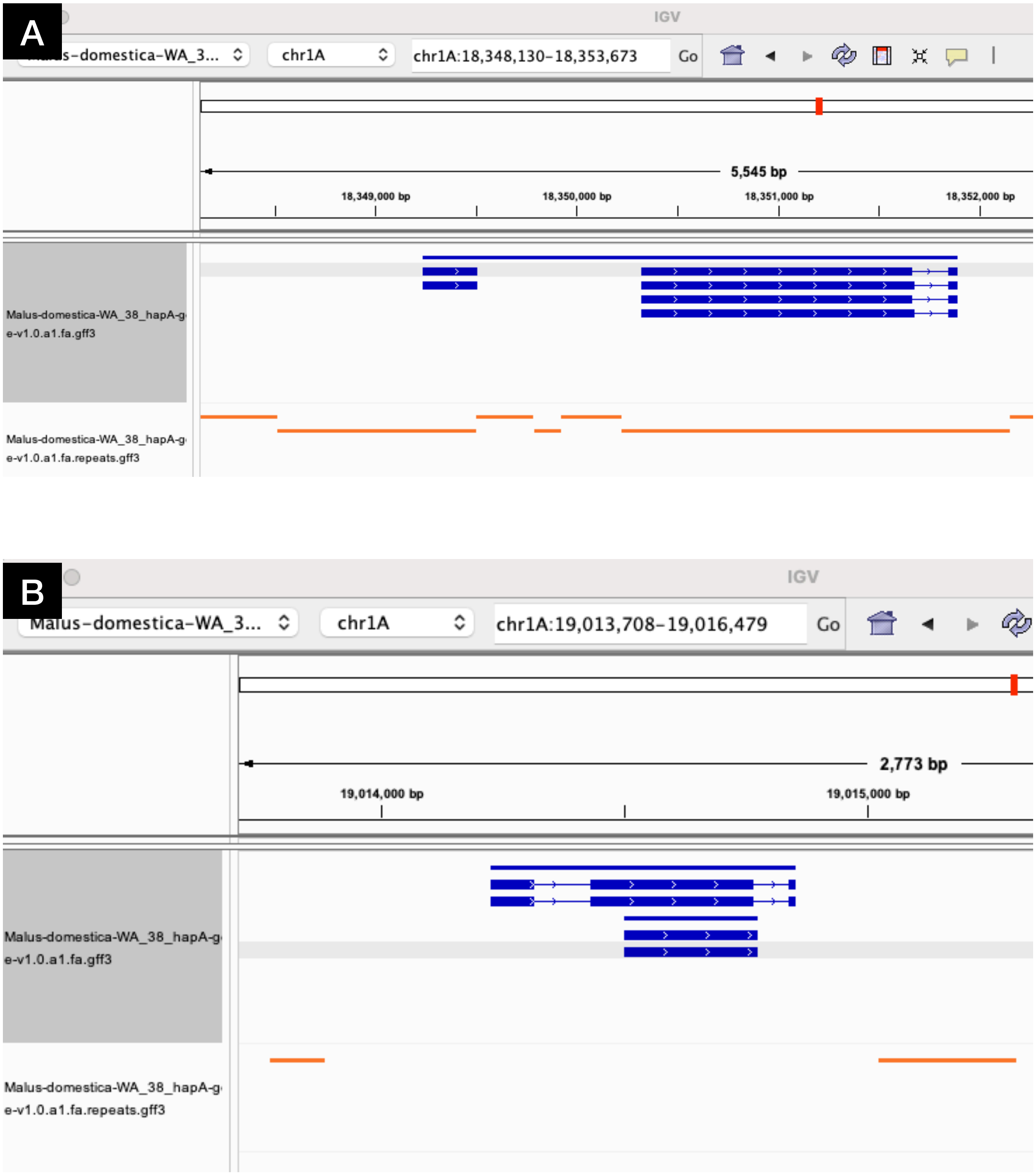

Figure S8. Riparian plot comparing 'WA 38' Haplotype A and B with 'Honeycrisp' Haplotype A and B and 'Golden Delicious' (GDDH13) genomes by physical location.

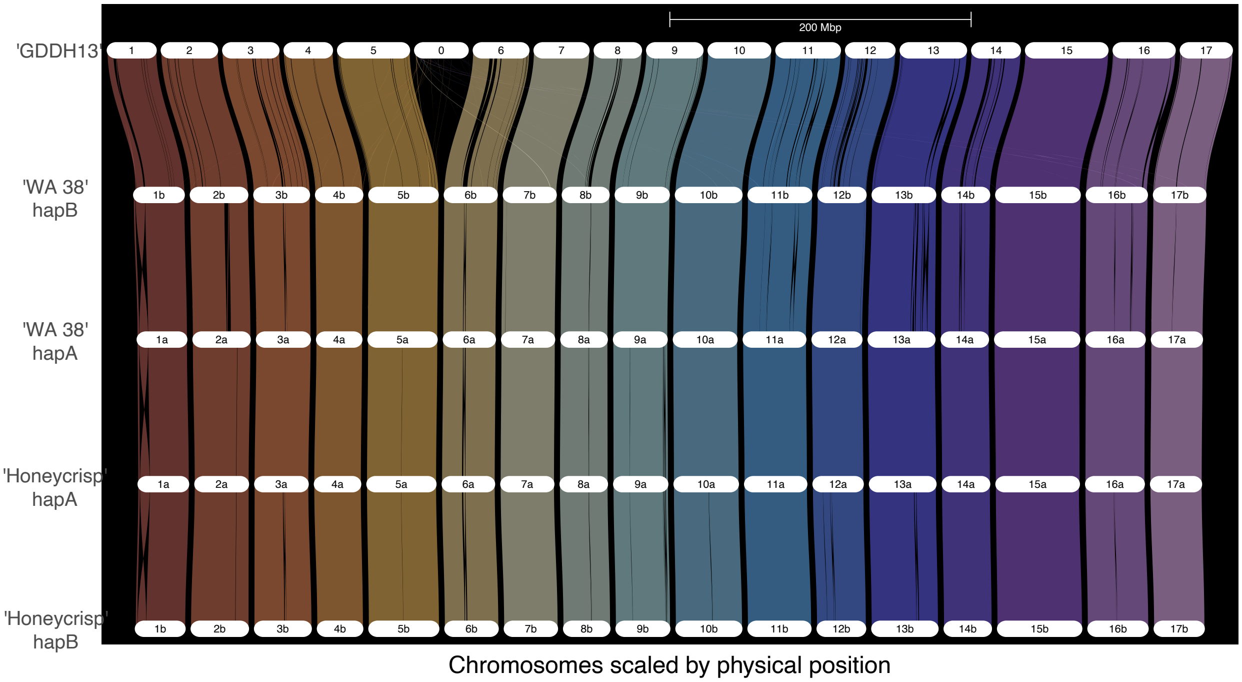

Figure S9. CROG analysis using ‘WA 38’ Subset 1 (**A** & **C**) and Subset 3 (**B** & **D**). **A** & **B** are CROG gene count cluster maps. Each row represents a CROG and each column represents a genomes. Color indicates the number of genes in each cell relative to the row average (z-score). Warmer color indicates more genes. Cooler color indicates fewer genes. The darker a color, the closer the value is to the row average. Green boxes highlights shared ‘cold’ orthogroups among genomes released from the same publication, or analyzed by the same research groups/ individual. **C** & **D** are CROG gene count z-score box plots summarizing z-score distribution of CROG gene counts in selected pome fruit genomes. Genome and annotation abbreviations can be found in Supplemental Table 1.

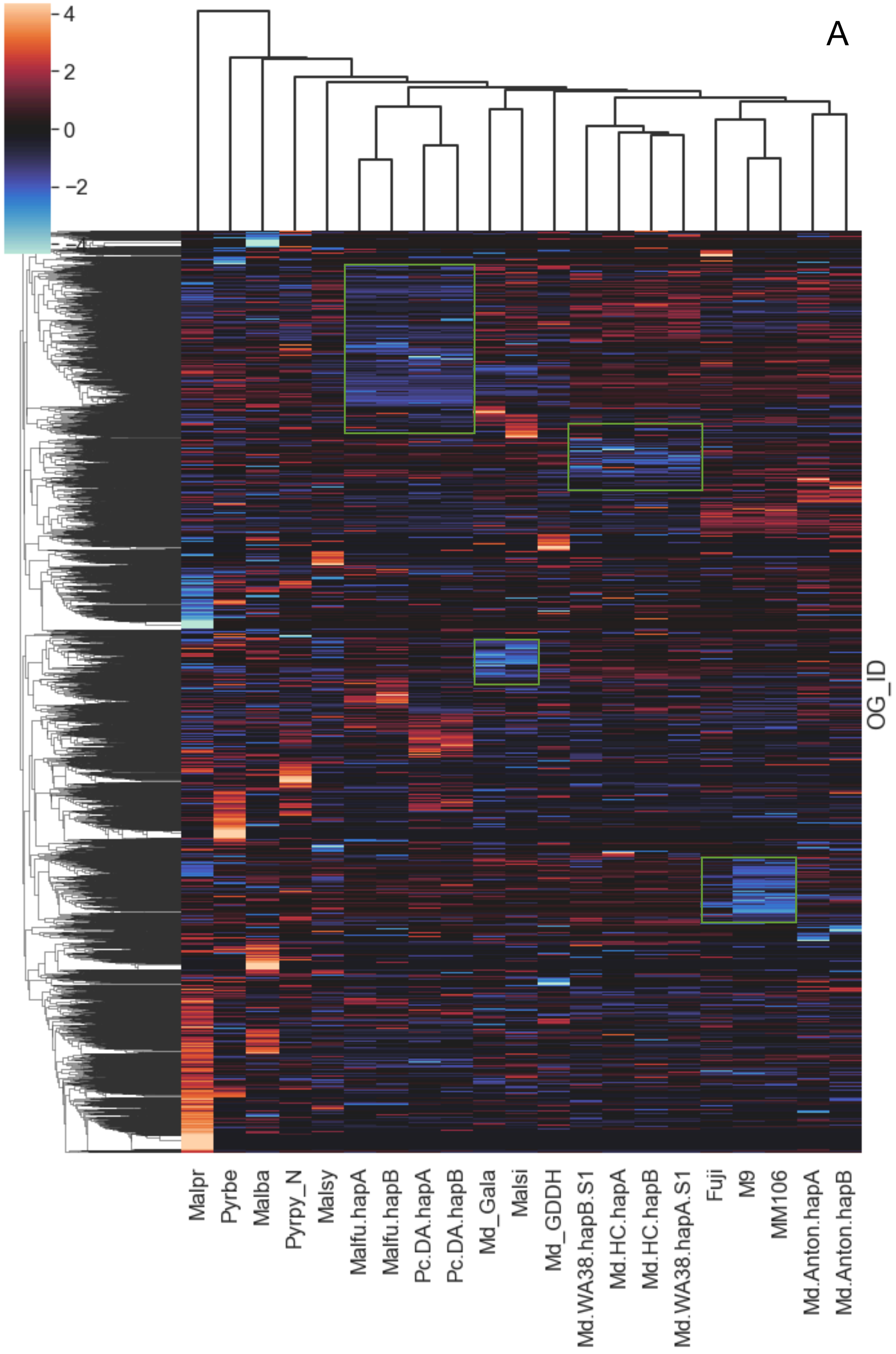

A
4
2
0
−2
−4
OG_ID
Malpr
Pyrbe
Malba
Pyrpy_N
Malsy
Malfu.hapA
Malfu.hapB
Pc.DA.hapA
Pc.DA.hapB
Md_Gala
Malsi
Md_GDDH
Md.WA38.hapB.S1
Md.HC.hapA
Md.HC.hapB
Md.WA38.hapA.S1
Fuji
M9
MM106
Md.Anton.hapA
Md.Anton.hapB

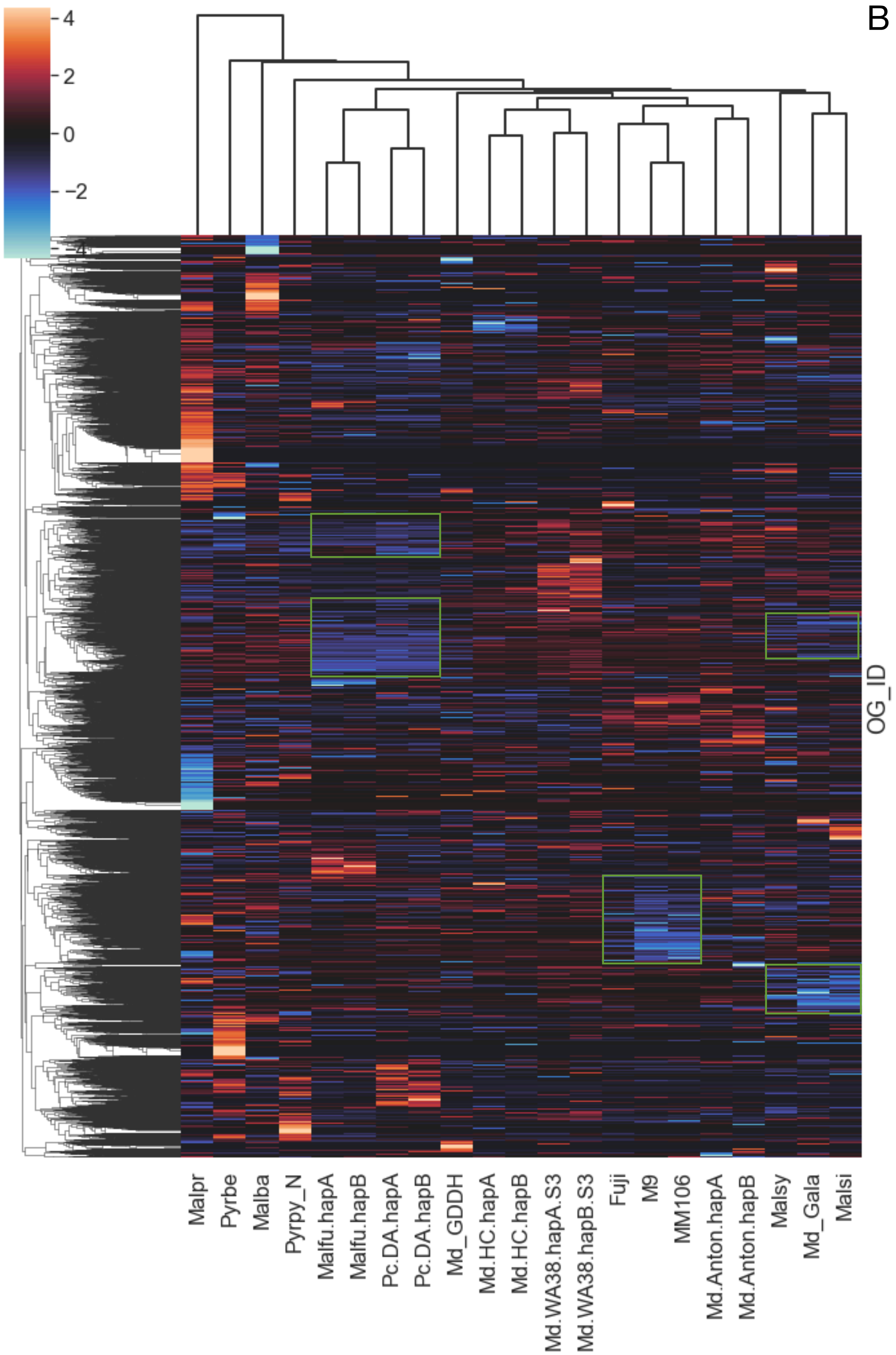
B
4
2
0
−2
−4
OG_ID
Malpr
Pyrbe
Malba
Pyrpy_N
Malfu.hapA
Malfu.hapB
Pc.DA.hapA
Pc.DA.hapB
Md_GDDH
Md.HC.hapA
Md.HC.hapB
Md.WA38.hapA.S3
Md.WA38.hapB.S3
Fuji
M9
MM106
Md.Anton.hapA
Md.Anton.hapB
Malsy
Md_Gala
Malsi

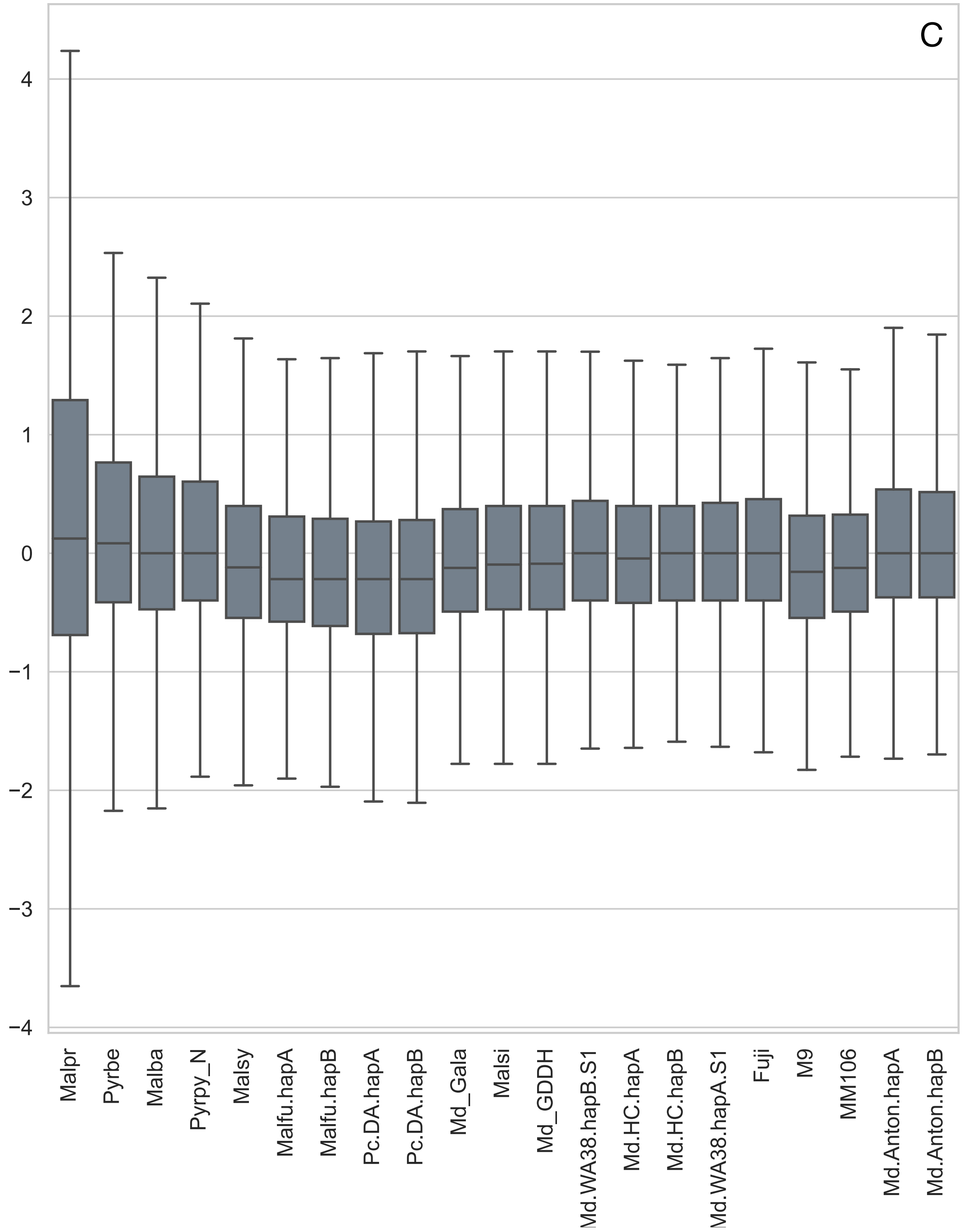
C
4
3
2
1
0
−1
−2
−3
−4
Malpr
Pyrbe
Malba
Pyrpy_N
Malsy
Malfu.hapA
Malfu.hapB
Pc.DA.hapA
Pc.DA.hapB
Md_Gala
Malsi
Md_GDDH
Md.WA38.hapB.S1
Md.HC.hapA
Md.HC.hapB
Md.WA38.hapA.S1
Fuji
M9
MM106
Md.Anton.hapA
Md.Anton.hapB

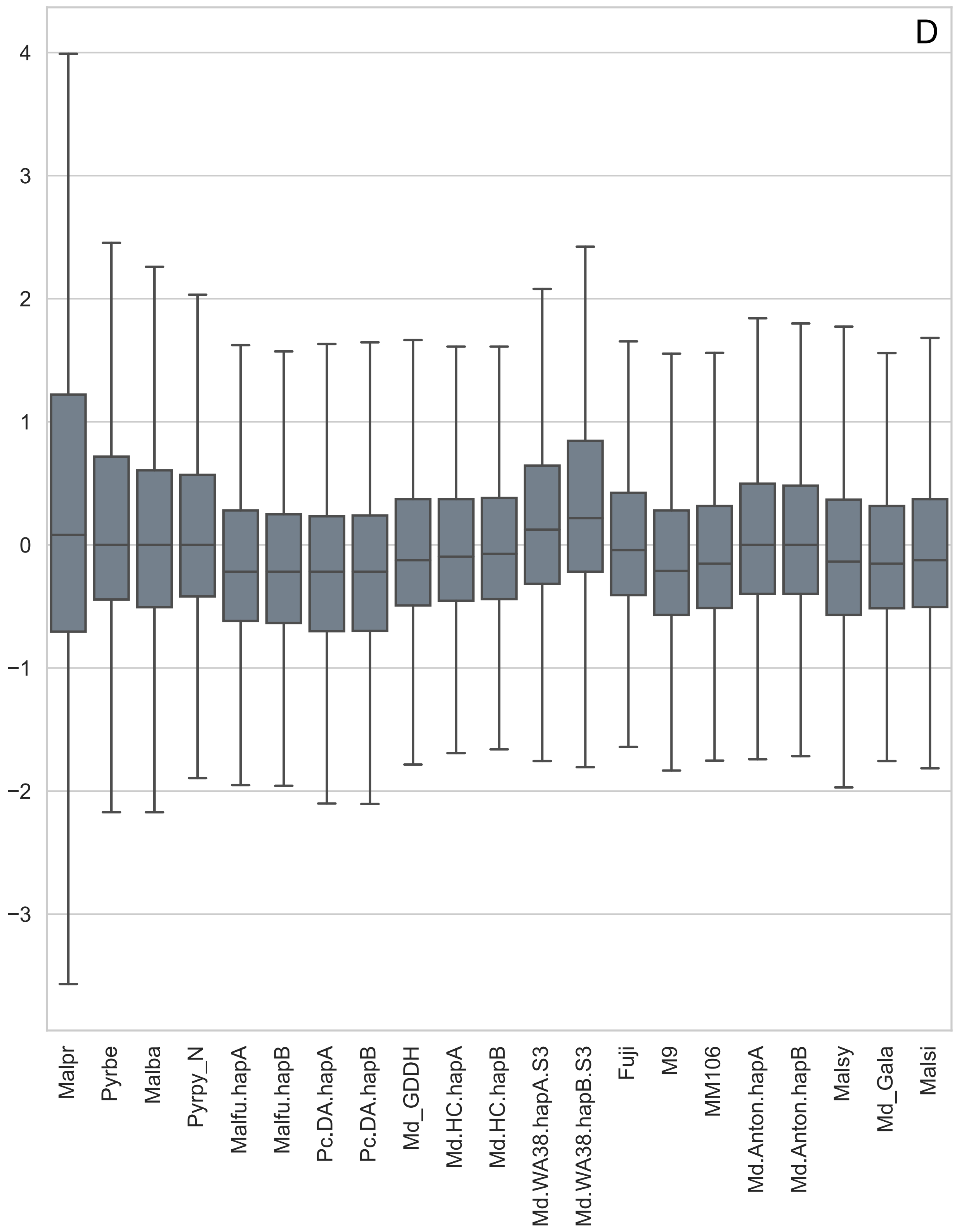
D
4
3
2
1
0
−1
−2
−3
Malpr
Pyrbe
Malba
Pyrpy_N
Malfu.hapA
Malfu.hapB
Pc.DA.hapA
Pc.DA.hapB
Md_GDDH
Md.HC.hapA
Md.HC.hapB
Md.WA38.hapA.S3
Md.WA38.hapB.S3
Fuji
M9
MM106
Md.Anton.hapA
Md.Anton.hapB
Malsy
Md_Gala
Malsi
